# Change of Direction Biomechanics in a 180-Degree Pivot Turn and the Risk for Noncontact Knee Injuries in Youth Basketball and Floorball Players

**DOI:** 10.1177/03635465211026944

**Published:** 2021-07-20

**Authors:** Mari Leppänen, Jari Parkkari, Tommi Vasankari, Sami Äyrämö, Juha-Pekka Kulmala, Tron Krosshaug, Pekka Kannus, Kati Pasanen

**Affiliations:** † Tampere Research Center of Sports Medicine, UKK Institute, Tampere, Finland; ‡ Tampere University Hospital, Tampere, Finland; § Faculty of Medicine and Health Technology, Tampere University, Tampere, Finland; ‖ Faculty of Information Technology, University of Jyväskylä, Jyväskylä, Finland; ¶ Motion Analysis Laboratory, Children’s Hospital, University of Helsinki and Helsinki University Hospital, Helsinki, Finland; # Oslo Sports Trauma Research Center, Department of Sports Medicine, Norwegian School of Sport Science, Oslo, Norway; ** Faculty of Kinesiology, Sport Injury Prevention Research Centre, University of Calgary, Calgary, Alberta, Canada; †† Alberta Children’s Hospital Research Institute, University of Calgary, Calgary, Alberta, Canada; ‡‡ McCaig Institute for Bone and Joint Health, University of Calgary, Calgary, Alberta, Canada; Investigation performed at the Tampere Research Center of Sports Medicine, UKK Institute, Tampere, Finland

**Keywords:** knee injuries, ACL injuries, risk factors, screening, biomechanics, team sports

## Abstract

**Background:**

Studies investigating biomechanical risk factors for knee injuries in sport-specific tasks are needed.

**Purpose:**

To investigate the association between change of direction (COD) biomechanics in a 180-degree pivot turn and knee injury risk among youth team sport players.

**Study Design:**

Cohort study; Level of evidence, 2.

**Methods:**

A total of 258 female and male basketball and floorball players (age range, 12-21 years) participated in the baseline COD test and follow-up. Complete data were obtained from 489 player-legs. Injuries, practice, and game exposure were registered for 12 months. The COD test consisted of a quick ball pass before and after a high-speed 180-degree pivot turn on the force plates. The following variables were analyzed: peak vertical ground-reaction force (N/kg); peak trunk lateral flexion angle (degree); peak knee flexion angle (degree); peak knee valgus angle (degree); peak knee flexion moment (N·m/kg); peak knee abduction moment (N·m/kg); and peak knee internal and external rotation moments (N·m/kg). Legs were analyzed separately and the mean of 3 trials was used in the analysis. Main outcome measure was a new acute noncontact knee injury.

**Results:**

A total of 18 new noncontact knee injuries were registered (0.3 injuries/1000 hours of exposure). Female players sustained 14 knee injuries and male players 4. A higher rate of knee injuries was observed in female players compared with male players (incidence rate ratio, 6.2; 95% CI, 2.1-21.7). Of all knee injuries, 8 were anterior cruciate ligament (ACL) injuries, all in female players. Female players displayed significantly larger peak knee valgus angles compared with male players (mean for female and male players, respectively: 13.9°± 9.4° and 2.0°± 8.5°). No significant associations between biomechanical variables and knee injury risk were found.

**Conclusion:**

Female players were at increased risk of knee and ACL injury compared with male players. Female players performed the 180-degree pivot turn with significantly larger knee valgus compared with male players. However, none of the investigated variables was associated with knee injury risk in youth basketball and floorball players.

Noncontact knee injury rates are alarmingly high and represent a significant concern in many pivoting sports, such as basketball^2,22^ and floorball.^29,36^ Severe knee injuries, such as anterior cruciate ligament (ACL) injury, will not only cause long-term absence from sports, but also devastating health problems. One of the serious consequences associated with acute knee injuries is early posttraumatic osteoarthritis.^21,41^ Furthermore, athletes with a previous knee injury frequently report negative health-related outcomes, such as knee-related symptoms, decreased quality of life, and higher body mass index.^40^ Although the effectiveness of multicomponent injury prevention programs to prevent knee injuries has been well-established,^5^ the incidence of acute noncontact knee injuries remains high in youth team sports.^27,28^

Identifying injury etiology is an important part of successful injury prevention.^37^ It is therefore important to investigate the risk factors that play a part in the occurrence of acute knee injuries. Acute noncontact knee injuries often occur during rapid dynamic movements such as change of direction (COD) maneuvers,^4,26^ which are associated with high external loads on the knee joint.^3^ The mechanism of ACL injury during sidestep cutting appears to include combinations of knee valgus, anterior tibial translation, and possibly internal rotation of the tibia when the knee is close to full extension.^12,13^ However, it is currently not known whether these or other biomechanical COD characteristics of athletes are associated with increased risk of future knee injuries, since prospective risk factor studies are lacking.

Previous prospective studies focusing on biomechanical risk factors for knee or ACL injuries have used baseline tests such as double- or single-leg vertical drop jump,^7,16,18,19^ single-leg squat,^32,33^ standing knee-lift,^20^ and core stability^43,44^ as screening tasks. These studies have identified a number of risk factors, including knee valgus loading,^7,10^ stiff landings,^18,19^ and altered hip control^20^ as well as decreased core proprioception^44^ and core stability.^43^ Nevertheless, evidence gathered from these investigations is conflicting,^16,34^ highlighting the need for improved tests. The use of more game-specific tasks, such as COD maneuvers, will generate substantially higher knee loads^14^ and are more closely related to actual injury situations. Thus, this task may be a better tool for investigating associations between COD biomechanics and knee injury.

The main aim of this study was to investigate the association between 180-degree pivot turn biomechanics and noncontact knee injury risk among youth basketball and floorball players. The secondary aim was to investigate the association between the pivot-turn biomechanics and noncontact ACL injury risk in female players. Furthermore, we aimed to describe potential sex differences in pivot-turn biomechanics.

## Methods

This prospective 12-month cohort study is a part of a large research project investigating risk factors of lower-extremity injuries in youth team sports. Detailed information on the study project is described elsewhere.^30^ The study has been conducted in accordance with the Declaration of Helsinki and was approved by the ethics committee of the Pirkanmaa Hospital District, Tampere, Finland (ETL-code R10169).

### Participants

We invited 9 basketball and 9 floorball teams from the 2 highest junior league levels from the Tampere region, Finland, to participate in the study. Players who were official members of the participating teams, and injury-free at baseline, were eligible for participation. Participating players completed a baseline questionnaire covering information about previous knee injuries and orthopaedic operations. Players with previous injuries were included if they were fully recovered when they entered the study. All participants signed a written informed consent form before the study, including parental consent for players aged <18 years.

**Figure 1.**
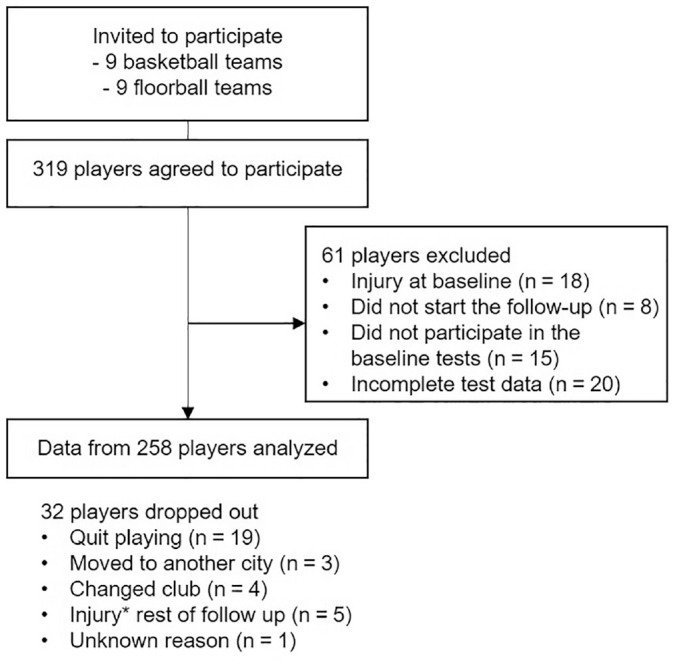
Flow of participants. The data from dropouts are included from the time they participated. *Injury other than acute noncontact knee injury.

A total of 319 players gave their consent of participation (Figure 1). Of them, 8 players stopped playing on participating teams before the follow-up, 18 players had an ongoing injury at baseline, 15 players did not participate in the test, and 20 players did not have 3 successful test trials (from either leg) and were excluded from the risk factor analyses. The reasons for excluding players with unsuccessful test trials included technical problems in 6 cases (ie, players were unable to do the COD correctly) and measurement-related issues, such as problems with markers, cameras, or force plates, in 14 cases. Test data from 1 leg only was included from 27 players. Of these, 9 players had technical difficulties and in 21 cases there were measurement-related reasons. Overall, data from 258 players (489 player-legs) were included in the analyses. After baseline screening testing, the players were followed prospectively for new knee injuries and game/practice exposure was documented for 12 months.

### Screening Test

At baseline, players performed a sport-specific, 180-degree pivot turn test in our 3-dimensional (3-D) motion analysis laboratory. The test was developed for the purpose of this study.^30^ The test was designed by a national floorball team coach (K.P.). In addition, top-level basketball coaches were consulted before the study to confirm that the test would work for youth basketball players as well.

All players wore shorts and indoor sports shoes, and female players additionally wore sports bras. We measured height and body mass as well as knee and ankle joint widths before the test. One trained physical therapist placed the reflective markers on all players. Bilateral placement of markers was carried out according to a Plug-in Gait full body model (Vicon) and included both upper body and lower body (on the shoe over the second metatarsal head and over the posterior calcaneus, lateral malleolus, lateral shank, lateral femoral epicondyle, lateral thigh, anterior superior iliac spine, and posterior superior iliac spine). Kinematic and kinetic calculations were performed using the proximal segment reference plane.

Before the test, each player performed a 5-min cycling warm-up. The players were allowed to perform 1 to 3 practice trials. The recorded trials were accepted if the player performed the test as instructed with maximal approach speed and full effort in the turn (visually evaluated), the entire foot landed on the force plate (each foot on separate force plates), and the markers stayed firmly on the player’s skin throughout the trial.

**Figure 2.**
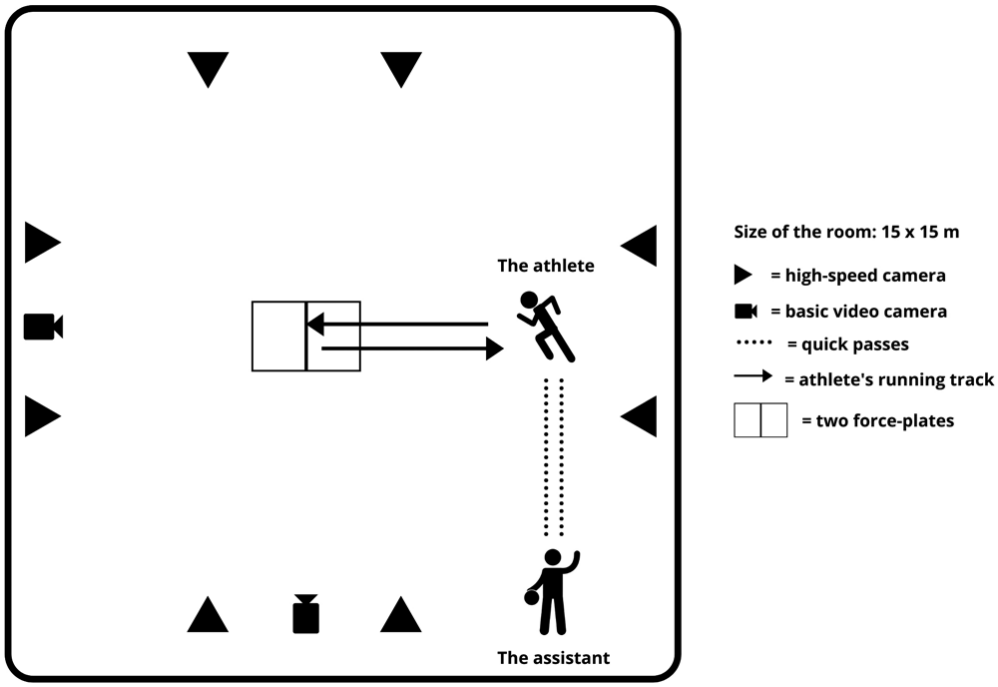
Setup for the change of direction test.

**Figure 3.**
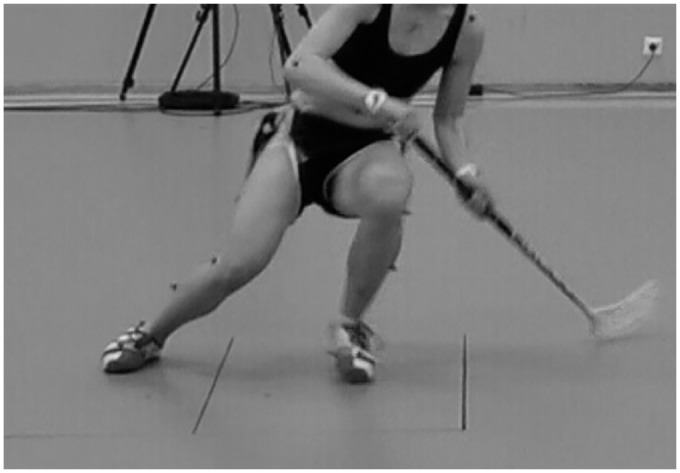
A floorball player performing the 180-degree pivot turn on the force plates.

Initially, the player started in a proper playing posture. Subsequently, the player received a quick pass from a study assistant and passed the ball back to the assistant (1-touch pass) (Figure 2). Then the player accelerated for 4 meters on her/his lateral direction, performed a quick 180-degree pivot turn on the force plates (Figure 3), and returned as fast as possible to the starting point where she/he received and passed the ball again (see Supplementary Video, available online).

### Motion Data Collection

Eight infrared, high-speed cameras (Vicon T40; Vicon) and 2 force platforms (BP6001200; AMTI) were used to record marker positions and ground-reaction force data synchronously at 300 and 1500 Hz, respectively. We conducted a static calibration trial before the test to determine the anatomic segment coordinate systems. Marker trajectories were identified using Vicon Nexus software. Both movement and ground-reaction force were filtered using a fourth-order Butterworth filter with cutoff frequencies of 15 Hz.^15^ The contact phase was defined as the period when the unfiltered ground-reaction force exceeded 20 N.

### Data Analysis

We analyzed kinetics and kinematics of the left knee (ie, outside leg) during right turns and kinetics and kinematics of the right knee during left turns. We collected 3 valid trials from both sides. The mean peak value of the trials was used in the analyses. To limit the number of variables tested, we aimed to focus on biomechanical variables of the knee during the contact phase of the 180-degree pivot turn. In addition, we used 1 variable to describe lateral trunk motion during the pivot turn. The predefined biomechanical variables included peak vertical ground-reaction force, peak trunk lateral flexion angle, and peak knee flexion and valgus angles, as well as peak knee flexion, abduction, and internal/external rotation moments. Knee joint angles and moments were calculated about an orthogonal axis system located in the proximal segment of a joint (thigh), whereas trunk movements were expressed in the laboratory coordinate system.

An open-source Python wrapping of Biomechanical ToolKit platform (BTK 0.3) and a custom Python Version 3.7.4 script were used for reading and modifying the kinematic and kinetic 3-D trajectories and force plate signals from the c3d files. Moreover, the standard open-source Python library for scientific computing (NumPy 1.16.5) was utilized in the extraction of the biomechanical variables from the imported raw time-series data vectors.

### Injury and Exposure Registration

After the baseline tests, a 12-month follow-up was conducted during which acute time-loss knee injuries as well as exposure data were recorded.

A study physician was responsible for collecting the injury data. The physician contacted the teams once a week to check for possible new injuries. After each reported injury, the study physician performed a structured interview with the injured player using an injury report form. Practice- and game-related acute noncontact (ie, no direct contact to the knee) joint and ligament injuries of the knee area were registered. We recorded an injury if the player was unable to fully participate in a team practice session or game during the next 24 hours.^8^ We recorded a new ACL injury if the injury was a noncontact ACL rupture confirmed by magnetic resonance imaging (MRI) that occurred during a team practice session or game.

During the follow-up, team coaches recorded individual player participation in practices and games in a team diary. Coaches completed these forms after each practice and game. We collected team diaries from the coaches on a monthly basis.

### Statistical Methods

Descriptive statistics were reported as the mean and standard deviation. Group differences were analyzed using Mann-Whitney *U* test (SPSS Version 25.0; IBM).

Separate Cox mixed-effects regression models^35^ were generated for each candidate risk factor. Hazard ratios with 95% CIs were calculated using R Version 3.5.1 (R Foundation for Statistical Computing). A new acute noncontact time-loss knee injury was the primary outcome and a new noncontact ACL injury was the secondary outcome of the analysis. The models included the monthly exposure time from the start of the follow-up until the first injury or the end of the follow-up. In addition, all models included sports club and leg as random effects. Both unadjusted and adjusted models were analyzed. In the adjusted models, we included potential confounders that might influence the risk of injury. First, potential confounders such as sex, age, height, body mass, sport, dominant leg, participation in adult league level games, and previous acute knee/ACL injury were included in a regression model. In the analyses of knee injury risk factors, we defined previous injury as any acute knee injury affecting either of the legs, and in the analyses of ACL injuries, we defined the previous injury as an MRI-confirmed ACL injury affecting either of the legs. Variables with a *P* value > .2 were removed 1 by 1. Following the guideline of 10 events per 1 variable,^31^ the variables with the lowest *P* values were included in the final models. Because all ACL injuries in our study occurred in female players, we analyzed ACL injury risk factors by including only female players.

## Results

Altogether 258 players (130 basketball and 128 floorball players) were included in the study (Table 1). Complete data for the baseline test and injury and exposure registration were obtained from 489 player-legs. A total of 18 new noncontact knee injuries were registered during the follow-up (incidence rate 0.3 injuries/1000 hours of exposure). Female players sustained 14 knee injuries and male players 4 (incidence rates 0.6 and 0.1 injuries/1000 hours of exposure, respectively). Female players had a significantly higher rate of knee injuries compared with male players (incidence rate ratio, 6.2; 95% CI, 2.1-21.7). Of the knee injuries, 8 were ACL injuries and all of them affected female players. The other injuries included 3 knee hyperextension injuries, 2 lateral collateral ligament strains, a medial collateral ligament strain, a patellar dislocation, and 3 unspecified acute knee injuries.

**Table 1:** Characteristics of the Participants (N = 258)*^a^*

	Basketball (n = 130)		Floorball (n = 128)	
	Male (n = 70)	Female (n = 60)	Male (n = 79)	Female (n = 49)
Age, y	15.3 ± 1.9	14.5 ± 1.3	16.9 ± 1.4	17.5 ± 2.0
Height, cm	180.5 ± 9.5	168.4 ± 6.6	177.5 ± 6.1	166.5 ± 5.9
Body mass, kg	70.0 ± 13.4	60.8 ± 8.6	68.9 ± 7.9	62.2 ± 7.7
BMI	21.3 ± 3.0	21.4 ± 2.8	21.9 ± 2.2	22.5 ± 2.7
Playing experience, y	7.2 ± 3.1	6.7 ± 2.6	8.7 ± 2.9	7.5 ± 2.5
Practice exposure, h	17,493	10,273	20,620	11,199
Game exposure, h	532	457	829	505
New knee injury, n	3	4	1	10
New ACL injury, n	0	1	0	7

a Data are reported as n or mean ± SD. ACL, anterior cruciate ligament; BMI, body mass index.

Of the 18 players who suffered a knee injury during the follow-up, 5 players had sustained a previous acute knee injury: 2 players had an ipsilateral reinjury and 3 players had a contralateral reinjury. Out of 8 players with a new ACL injury, 3 had a previous ACL injury (1 ipsilateral and 2 contralateral reinjuries).

Female players displayed significantly larger mean peak knee valgus angles compared with male players (Table 2). Male players demonstrated significantly larger mean peak trunk lateral flexion angles compared with female players. In addition, significantly smaller mean peak abduction moments and internal rotation moments were observed in female players during the 180-degree pivot turn compared with male players.

**Table 2:** Test Results in Female and Male Players (n = number of legs)*^a^*

	Female (n = 203)	Male (n = 286)	*P* Value
Peak vertical GRF (N/kg)	20.5 ± 2.7	20.6 ± 2.8	.66
Peak trunk lateral flexion angle, deg*^b^*	1.6 ± 16.2	−3.6 ± 19.2	<.01*^c^*
Peak knee flexion angle, deg	62.6 ± 6.9	62.8 ± 7.8	.74
Peak knee valgus angle, deg	13.9 ± 9.4	2.0 ± 8.5	<.001*^c^*
Peak knee flexion moment, N·m/kg	2.3 ± 0.5	2.3 ± 0.7	.85
Peak knee abduction moment, N·m/kg	1.1 ± 0.4	1.2 ± 0.5	<.001*^c^*
Peak knee internal rotation moment, N·m/kg	0.4 ± 0.2	0.5 ± 0.2	<.001*^c^*
Peak knee external rotation moment, N·m/kg	0.2 ± 0.2	0.2 ± 0.1	.41

a Values are reported as mean ± SD. GRF, ground-reaction force.

b Negative value refers to ipsilateral and positive value to contralateral flexion of the trunk in relation to the outside leg.

c *P* < .05.

Group comparisons revealed no significant differences in test results in players with knee injury compared with the uninjured (Table 3).

**Table 3:** Test Results in Injured and Uninjured Legs*^a^*

	All Players			Male Players			Female Players			Female Players		
	Knee Injury (n = 18)	No Injury (n = 471)	*P* Value	Knee Injury (n = 4)	No Injury (n = 282)	*P* Value	Knee Injury (n = 14)	No Injury (n = 189)	*P* Value	ACL Injury (n = 8)	No Injury (n = 195)	*P* Value
Peak vertical GRF, N/kg	19.8 ± 1.8	20.6 ± 2.8	.24	21.7 ± 1.6	20.6 ± 2.9	.29	19.3 ± 1.5	20.6 ± 2.7	.05	18.7 ± 1.3	20.6 ± 2.7	.03
Peak trunk lateral flexion angle, deg	0.6 ± 18.8	−1.5 ± 18.2	.76	−6.5 ± 18.1	−3.5 ± 19.2	.70	2.7 ± 19.1	1.5 ± 16.0	.84	−2.8 ± 20.0	1.8 ± 16.1	.42
Peak knee flexion angle, deg	61.7 ± 6.6	62.8 ± 7.5	.52	65.5 ± 3.5	62.7 ± 7.8	.38	60.7 ± 6.9	62.8 ± 6.9	.20	63.3 ± 7.1	62.6 ± 6.9	.89
Peak knee valgus angle, deg	10.4 ± 11.4	6.8 ± 10.6	.20	5.1 ± 11.4	2.0 ± 8.4	.54	11.9 ± 11.4	14.0 ± 9.2	.38	16.1 ± 12.6	13.8 ± 9.3	.52
Peak knee flexion moment, N·m/kg	2.2 ± 0.5	2.3 ± 0.6	.55	2.6 ± 0.5	2.3 ± 0.7	.24	2.1 ± 0.4	2.3 ± 0.6	.12	2.1 ± 0.4	2.3 ± 0.6	.50
Peak knee abduction moment, N·m/kg	1.2 ± 0.5	1.2 ± 0.5	.94	1.5 ± 0.7	1.2 ± 0.5	.41	1.1 ± 0.5	1.1 ± 0.4	.96	1.3 ± 0.5	1.1 ± 0.4	.11
Peak knee internal rotation moment, N·m/kg	0.4 ± 0.2	0.5 ± 0.2	.26	0.7 ± 0.3	0.5 ± 0.2	.24	0.4 ± 0.1	0.4 ± 0.2	.39	0.4 ± 0.1	0.4 ± 0.2	.78
Peak knee external rotation moment, N·m/kg	0.2 ± 0.1	0.2 ± 0.2	.66	0.2 ± 0.1	0.2 ± 0.1	.76	0.2 ± 0.1	0.2 ± 0.2	.85	0.2 ± 0.1	0.2 ± 0.2	.93

a Values are presented as mean ± SD. GRF, ground-reaction force.

The results from regression analyses for unadjusted and adjusted models are presented in Table 4. In unadjusted models, increased knee valgus angle had a significant association for knee injury risk (hazard ratio for 1 degree increase in knee valgus: 1.04; 95% CI, 1.00-1.08; *P* = .03). However, in adjusted models, none of the investigated variables were associated with the risk of knee or ACL injury.

**Table 4:** Association Between Biomechanical Variables and Knee and ACL Injury Risk*^a^*

	Knee Injuries (n = 18)		ACL Injuries (n = 8)*^b^*	
	Unadjusted Model	Adjusted Model	Unadjusted Model	Adjusted Model
Peak vertical GRF, N/kg	0.90 (0.90-1.08)	0.92 (0.76-1.11)	0.70 (0.49-1.00)	0.68 (0.46-1.01)
Adjustment factors		Sex, male 0.10 (0.03-0.33)		Age 1.46 (1.06-2.00)
		Playing years 1.30 (1.08-1.56)		
Peak trunk lateral flexion angle, deg	0.99 (0.97-1.02)	1.00 (0.98-1.03)	1.01 (0.97-1.05)	1.02 (0.98-1.06)
Adjustment factors		Sex, male 0.10 (0.03-0.33)		Age 1.52 (1.08-2.14)
		Playing years 1.31 (1.08-1.58)		
Peak knee flexion angle, deg	0.99 (0.93-1.05)	0.99 (0.93-1.05)	1.01 (0.92-1.11)	1.01 (0.91-1.11)
Adjustment factors		Sex, male 0.10 (0.03-0.33)		Age 1.50 (1.05-2.15)
		Playing years 1.30 (1.08-1.57)		
Peak knee valgus angle, deg	1.04 (1.00-1.08)*^c^*	0.98 (0.93-1.02)	1.01 (0.95-1.08)	1.00 (0.93-1.07)
Adjustment factors		Sex, male 0.07 (0.02-0.28)		Age 1.50 (1.05-2.16)
		Playing years 1.35 (1.10-1.64)		
Peak knee flexion moment, N·m/kg	0.95 (0.46-1.98)	0.75 (0.32-1.73)	0.54 (0.13-2.22)	0.37 (0.07-1.84)
Adjustment factors		Sex, male 0.10 (0.03-0.32)		Age 1.54 (1.10-2.16)
		Playing years 1.31 (1.09-1.58)		
Peak knee abduction moment, N·m/kg	0.99 (0.38-2.59)	0.93 (0.33-2.61)	2.29 (0.46-11.5)	1.24 (0.21-7.44)
Adjustment factors		Sex, male 0.10 (0.03-0.33)		Age 1.48 (1.01-2.17)
		Playing years 1.31 (1.08-1.58)		
Peak knee external rotation moment, N·m/kg	1.99 (0.07-55.2)	1.88 (0.07-51.1)	1.62 (0.01-300.7)	2.78 (0.02-482.8)
Adjustment factors		Sex, male 0.11 (0.03-0.34)		Age 1.51 (1.07-2.14)
		Playing years 1.31 (1.09-1.57)		
Peak knee internal rotation moment, N·m/kg	0.38 (0.03-4.43)	1.26 (0.07-10.3)	0.45 (0.01-26.8)	0.70 (0.00-21.8)
Adjustment factors		Sex, male 0.10 (0.03-0.34)		Age 1.53 (1.07-2.19)
		Playing years 1.30 (1.08-1.57)		

a Values are reported as Cox hazard ratios (95% CI). ACL, anterior cruciate ligament; GRF, ground-reaction force.

b Only female players included.

c *P* value < .05.

## Discussion

A large proportion of the acute knee injuries in basketball and floorball occur during COD maneuvers. Hence, this study aimed to investigate whether the COD technique with high knee loading during a 180-degree pivot turn is associated with future knee injury risk. Against our hypothesis, none of the investigated biomechanical factors during the contact phase of the outside leg during the 180-degree pivot turn were found to be risk factors for knee injuries in youth team sport players.

A high incidence of knee injuries in youth team sport players has been reported in several studies,^1,2,28^ and female players, in particular, are at increased risk of knee injury.^25^ Female athletes have been suggested to commonly display a so-called “ligament dominance” where external loads are absorbed by ligaments instead of muscles.^9^ Currently, only 1 prospective study has been able to identify knee abduction loading as a risk factor for knee injuries using a vertical drop jump test.^10^ Similar studies with higher statistical power^16,19^ have not been able to replicate those findings. However, as the loads are far greater in COD maneuvers compared with drop jumps,^14^ it seems more likely that we could detect knee load–related risk factors in such maneuvers. However, this was not the case in the current study. In our study, we made the COD test more sport-specific by adding passing and receiving a ball before and after the 180-degree pivot turn. Nevertheless, we did not include unanticipated elements, which might have yielded even higher knee loading.^38,39^ Future studies are suggested to investigate biomechanical risk factors during sport-specific context.

The female players in our study displayed substantially larger peak knee valgus angles compared with their male counterparts, but on the other hand, the knee abduction loading was higher in male players, even though the vertical ground-reaction force and knee flexion moments were virtually identical between sexes. Thus, it seems that male players were able to control these loads better than female players. Importantly, knee abduction loads can be counteracted by muscle efforts of both quadriceps and hamstring muscles,^10^ thus we cannot determine how great the loads were on the ligaments based on our measurements.

In our study, we observed large knee valgus angles in injured players (10.4°) and notably large valgus angles (16.1°) in female players with ACL injuries, but the difference between the injured and uninjured players was not significant, possibly due to sample size limitations. Interestingly, we also observed knee abduction moments of 1.3 N·m/kg in players with ACL injuries versus 1.1 N·m/kg in players without ACL injuries, corresponding to a 20% difference, which would likely have clinical importance. However, again there was no statistical difference between groups; thus, new studies must be conducted to confirm whether knee abduction loading would show up as a risk factor in a larger study sample.

Increased trunk lateral flexion over the support leg has been shown to increase knee loading during cutting maneuvers^6,11^ and together with knee valgus also increase the risk of knee injury in female athletes.^7^ Although female players suffered most of the knee injuries and all ACL injuries in our study, female players displayed greater contralateral trunk motion during the COD task in contrast to male players. Lateral trunk flexion does not therefore seem to influence injury risk in our current population.

Previous modeling and simulation studies have suggested that sagittal plane knee loading plays an important role in the ACL injury mechanism.^38,39,42^ Moreover sagittal plane stiffness has also been identified as a risk factor for ACL injuries.^19^ In this study, we did not find any associations between sagittal plane variables and injury risk. This may indicate that sagittal plane factors in such preplanned screening tasks are not predictive of the risk for a future injury. Still, given that sagittal plane loading contributes to ACL loading, it may still be wise to consider such loads when developing strategies to reduce injury risk.

The major strength of our study is that it includes a prospective study setting using dynamic task simulating real COD maneuvers. To our knowledge, no previously published prospective study has investigated knee biomechanics during cutting maneuvers as a risk factor for knee injuries.

Nevertheless, some limitations exist. The main weakness of our study is a relatively low number of injuries. Although our results were possibly affected by low statistical power, it is likely that factors with strong associations would have been detected. Future studies should aim to recruit larger number of players and conduct longer follow-ups to investigate risk factors with better precision. In addition, we tested players only at the baseline of the study, and we acknowledge that biomechanics may change in growing athletes. Furthermore, we could not objectively measure players’ level of effort, and hence the technique might not reflect the true game situation.

Although 3-D motion analysis is considered the gold standard, errors may occur. Most commonly errors originate from marker placement^24^ and skin artifacts.^17^ To minimize the errors in marker placement, we used 1 physical therapist who was trained to place the markers uniformly and according to our protocol. Furthermore, reliability of sport-specific cutting tasks has been assessed earlier and shown to have good within- and between-session reliability, and adequate reliability can be obtained with using 3 trials only, supporting the use of these methods in our study.^23^

In conclusion, none of the investigated biomechanical variables of the 180-degree pivot-turn test were associated with knee injury risk in youth male and female team sports players, but due to the limited study size, only strong associations would be detected. All ACL injuries occurred in female players, and excessive knee valgus movement was far more common in youth female players compared with their male counterparts, possibly contributing to the sex difference in risk of ACL injury. The COD test used in the current study is not useful to identify youth athletes at high risk for knee injury.
